# THE ROLE OF MILD COGNITIVE IMPAIRMENT AND MARITAL STATUS ON LONELINESS AND INTRUSIVE THOUGHTS IN DAILY LIFE

**DOI:** 10.1093/geroni/igad104.1330

**Published:** 2023-12-21

**Authors:** Karina Van Bogart, Christopher Engeland, Dakota Witzel, Jennifer Graham-Engeland

**Affiliations:** Penn State University, State College, Pennsylvania, United States; Pennsylvania State University, University Park, Pennsylvania, United States; Penn State University, State College, Pennsylvania, United States; Pennsylvania State University, State College, Pennsylvania, United States

## Abstract

Loneliness is associated with maladaptive cognitions (e.g., rumination and intrusive thoughts) among young adults, but less is known about this association among older adults. Experiencing loneliness and intrusive thoughts throughout daily life may disrupt emotion regulation and increase risk for cognitive decline during later life. Moreover, social and cognitive factors, including marital status and mild cognitive impairment (MCI), may be protective or exacerbating factors that moderate these associations. For example, having close relationships (e.g., being married) is associated with better mental health and overall well-being for older adults and intrusive thoughts have been associated with poorer cognitive performance. We used multilevel modeling to examine the association between momentary loneliness and intrusive thoughts among older adults and whether MCI and marital status moderated this association. Participants were 316 diverse older adults without dementia (40% Black; 13% Hispanic, mean age = 77.45 years, 67% women) living in the Bronx, NY who completed ecological momentary assessments 5 times daily for 14 consecutive days. There was a significant three-way interaction (β = -0.175, p < 0.05) such that the association between momentary loneliness and intrusive thoughts was strongest for those with MCI who were not married and weakest for those with MCI who were married. Covariates included race/ethnicity, years of education, age, and gender. These findings suggest that for those who have MCI, being married may be a protective factor against moments of loneliness and intrusive thoughts. For those who are not married, tailoring interventions to accommodate MCI status may be worthwhile.

